# Identifying Mobile Health Engagement Stages: Interviews and Observations for Developing Brief Message Content

**DOI:** 10.2196/15307

**Published:** 2020-09-22

**Authors:** Kara Burns, Rebekah Nicholas, Amanda Beatson, Marianella Chamorro-Koc, Alethea Blackler, Udo Gottlieb

**Affiliations:** 1 School of Advertising, Marketing and Public Relations QUT Business School Queensland University of Technology Brisbane, QLD Australia; 2 QUT Design Lab Creative Industries Faculty Queensland University of Technology Brisbane, QLD Australia

**Keywords:** mobile health, text messaging, social media, mobile phone, health communication

## Abstract

**Background:**

Interest in mobile health (mHealth) has increased recently, and research suggests that mHealth devices can enhance end-user engagement, especially when used in conjunction with brief message content.

**Objective:**

This research aims to explore the *stages of engagement* framework for mHealth devices and develop a method to generate brief message content to promote sustained user engagement. This study uses the framework by O’Brien and Toms as a point of departure, where engagement is defined as the uptake or the use of an mHealth device. The framework is a linear repeatable process, including *point of engagement*, *period of engagement*, *disengagement*, and *re-engagement*. Each stage is characterized by attributes related to a person’s technology experience. Although the literature has identified stages of engagement for health-related technology, few studies explore mHealth engagement. Furthermore, little research has determined a method for creating brief message content at each stage in this engagement journey.

**Methods:**

Interviews and observations from 19 participants who used mHealth technologies (apps, devices, or wellness websites) in a solo capacity were recruited for sample group 1. In sample group 2, interviews, and observations from 25 participants using mHealth technologies in a group capacity through the Global Corporate Challenge were used. These samples were investigated at 3 time points in both research contexts. The results underwent deductive-inductive thematic analysis for the engagement stages’ framework and attributes.

**Results:**

In addition to the 4 stages identified by O’Brien and Toms, 2 additional stages, self-management and limited engagement, were identified. *Self-management* captures where users had disengaged from their technology but were still engaged with their health activity. *Limited engagement* captures where group mHealth users had minimal interaction with their mHealth technology but continued to engage in a group fitness activity. The results revealed that mHealth engagement stages were nonlinear and embedded in a wider engagement context and that each stage was characterized by a combination of 49 attributes that could be organized into 8 themes. Themes documented the total user experience and included technology usability, technology features, technology aesthetics, use motivations, health awareness, goal setting, social support, and interruptions. Different themes were found to have more relevance at different engagement stages. Knowing themes and attributes at all engagement stages allows technology developers and health care professionals to generate relevant brief message content informed by a person-centered approach.

**Conclusions:**

This research extends an existing engagement stages framework and identifies attributes and themes relevant to mHealth technology users’ total user experience and incorporates concepts derived from health, business studies, and information systems literature. In addition, we offer a practical 5-step process based on a person-centered approach to develop mHealth technology brief message content for sustained engagement.

## Introduction

### Mobile Health

Since 2013, mobile health (mHealth) or the use of mobile computing and communication technologies in health care, public health, and personal wellness has gained significant interest, including the use of SMS through mobile devices. This is because of efforts to engage patients [1] and improve health outcomes at lower costs [2]. mHealth is considered to be a subset of the broader eHealth movement, which involves the digitization of health care processes [3]. Although eHealth and mHealth are potentially transformative for health care users, population health, and health care systems, the benefit of the significant economic investment in these modalities is undergoing scrutiny. For example, a 2011 review of the billions of dollars spent on eHealth systems globally has shown no evidence that implementing these technologies reduces health service costs [4].

When investigating clinical impact, early randomized control trials focused on mHealth feasibility. Current research has shown mHealth to be effective for improving health outcomes, especially when using brief messages such as those delivered by SMS through personal devices (eg, smartphones). Brief messages are cost-effective and can be delivered en masse in low-resource areas and can be personalized to target specific populations [5]. For example, women in low- and middle-income countries who received SMS support during pregnancy showed reduced chances of infant morbidity or mortality [6]. This is congruent with other SMS mHealth initiatives that have shown an increase in smoking cessation [7], improvements in the uptake of sexual health services using SMS reminders compared with controls [8], and reduced viral loads of patients with HIV when reminded to take antiretroviral medication [9].

For mHealth interventions that include a communication component, message content is critical to the effectiveness of the interventions. For example, in a 24-month randomized clinical trial for weight loss, targeted feedback from health care providers by SMS was shown to improve engagement behavior in self-reported energy and fat intake [10]. An important aspect of this study was that messages were personalized using the individual feedback provided by participants. Communication between health care providers and patients holds an immediate opportunity for improved clinical outcomes and patient engagement, emphasizing the importance of identifying suitable message content for this communication [5]. However, one of the key challenges faced by these health care services is how to understand patterns of engagement and keep users engaged [11].

### mHealth Engagement

mHealth engagement is complex because of numerous definitions and the technology straddling the disciplines of health care, information systems, and business studies. In this research, engagement is defined as the uptake or use of an mHealth device [12]; however, limiting the exploration of mHealth to one discipline skews our understanding of the phenomena, resulting in a partial view of the impact of the technology and engagement.

Through information systems research, the usability and design of technology play a key role in the uptake of mHealth [13]. It is recommended that the usability and visual appeal of apps should be taken into consideration from the beginning of the design process [13]; however, there is also an opportunity for it to be critiqued based on user feedback throughout the life of the mHealth intervention to support sustained engagement.

The business literature suggests that a user-centered experience is a key factor that contributes to engagement in service contexts, and this can also be applied to mHealth technology [14]. It is evident that there are different views on user experience. This paper defines the user experience as the total experience that a person obtains from all interactions with a health care provider and an mHealth device. This includes the value they receive [15-17]. The total user experience can be evidenced by end-user thoughts, feelings, and behaviors related to their technology use [18,19]. Value is critical to engagement and can be defined as a total assessment of the perceived cost [20] versus benefits [21,22]. If the effort to engage is greater than the total perceived value of the engagement, users may not be interested in an mHealth device.

**Figure 1:**
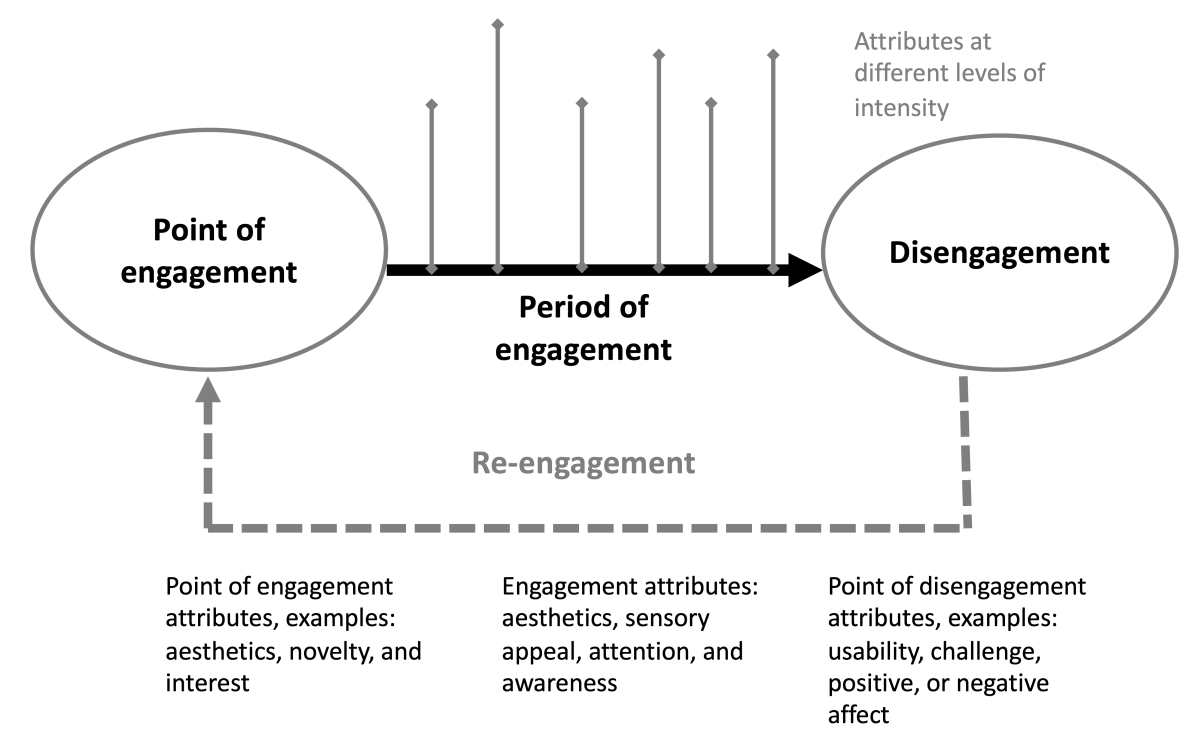
Proposed model of engagement by O'Brien and Toms (2008).

An interdisciplinary framework that incorporates health, information systems, and business does not exist in the present literature for mHealth engagement. However, O'Brien and Toms [12] developed a linear conceptual framework for defining technology user engagement that was applied in the context of web-based technology apps and incorporates attributes from both business and information systems. Through empirical inquiry, these authors demonstrated 4 stages of engagement, including point of engagement, period of engagement, disengagement, and re-engagement, that can be experienced in a linear sequence and repeated. Figure 1 provides the model of engagement stages, as proposed by O’Brien and Toms [12].

As illustrated earlier, the point of engagement is when the user encounters the technology for the first time, the period of engagement is the periodic use of the technology, disengagement is characterized by a period without using the technology, and re-engagement is a reintroduction of the technology to a previous user. Each stage in the process is characterized by themes and attributes. Attributes are the phenomena experienced by end users when engaging with mHealth technology, and these can be analyzed into themes. Attributes also signify the total user experience and are used as a baseline for exploration in this research. Importantly, this conceptualization provides a standard framework that can be flexibly applied in different contexts to explore the determinants of engagement in an mHealth context [23].

The framework of O'Brien and Toms has been explored in other digital health investigations, often underpinning studies that seek to conceptualize context-specific engagement [24]. Others simply apply the existing stages of the engagement framework [25,26]. Only one known study has explored additional stages by examining the notion of unengagement [11]. In this 2019 study of 486 health services’ users who accessed web-based health services [11], it was recognized that the *nonengagement* or *disengagement* were examples of unengagement. Thus, to date, the stages and attributes in the model by O’Brien and Toms require critique and further investigation, specifically within the context of mHealth, where exploration is limited.

With that in mind and using the framework by O’Brien and Toms as a departure point, this study aimed to develop a person-centered approach to generate relevant brief message content relating to engagement stages to promote sustained user engagement. When combined with users’ thoughts and observations of actual use, the framework by O’Brien and Toms can provide a way to investigate the stages of engagement for mHealth devices [12]. This is commensurate with the business literature and other health technology interventions that “demand a user-centered and iterative approach to development, using mixed methods and in-depth qualitative research to progressively refine the intervention to meet user requirements” [27]. Hence, this study asks, “How can knowledge about users’ mHealth device engagement stages inform the development of brief message content intended to promote sustained user engagement?”

## Methods

### Study Design

To gain rich insight, a qualitative approach was used to investigate the framework by O’Brien and Toms [28]. Qualitative studies allowed the researcher to observe or record behavior in its natural setting [28]. Interviews were used to explore participants’ state of mind in-depth [29], and face-to-face observations provided an understanding of participants’ behaviors [30].

In total, 2 sample groups were used in this research to deepen the understanding of mHealth technology use, avoiding the use of a single case to support findings. Participants were considered eligible if they had used an mHealth technology for at least 2 weeks before the interviews and observations (excluding pregnancy apps), were aged >18 years, were able to communicate effectively in English, and provided informed consent. Interviews and observations across the 2 sample groups were conducted until data saturation occurred [31]. This occurred in a total of 34 participants.

Samples 1 and 2 were contextually different, comparing mHealth engagement with solo (single person) technology use and mHealth engagement in a social (group) context. In both samples, a screening questionnaire, interviews, and structured face-to-face observations were undertaken. Questions included perceptions of use, value, behaviors, and engagement. Participants were asked about their program usage (either an individual program such as a tracking app for sample 1 or the group program for sample 2). Example questions included How has the program impacted your behavior toward wellness?, Can you please show me what you value about the program and explain why you have selected this?, and Why do you think your usage has dropped and/or increased? The full interview protocol is outlined in Multimedia Appendix 1. Ethics approval was obtained from the researcher’s home university (University Human Research Ethics Committee), and the research was conducted in line with standard ethical guidelines and the National Statement on Ethical Conduct in Human Research [32]. No incentives were offered in this study.

### Sample Group 1

Participants who used mHealth technologies (eg, mHealth apps or wellness websites) in an individual context for at least 2 weeks before the first interview were purposively recruited using a promotional flyer and snowball sampling in a large metropolitan city in Australia. Upon recruitment, participants attended in-depth confidential interviews and structured face-to-face observations using their mHealth technology in a private room or in a public location of their choice*.* Interviews and observations of the participant using the device were recorded by a researcher on a video camera. Participants were then invited to attend a second session 6 to 8 weeks after the first session and a final interview and observations at 8 to 10 months after the first session.

### Sample Group 2

Participants who used mHealth technologies in a group context were purposively recruited from the Global Corporate Challenge (GCC), a 100-day workplace team pedometer competition. The program is designed to promote workplace health and engagement around the world by encouraging employees to work in teams completing a minimum of 10,000 steps per day. After 2 to 4 weeks of engagement, participants attended in-depth confidential interviews and structured face-to-face observations using their mHealth technology in a private room or in a public location of their choice. These were recorded using a video camera by a researcher. All participants were invited to attend a second session 8-10 weeks after the first session and a final interview and observation 3-5 weeks after the GCC was complete.

### Data Analysis for Engagement Stages and mHealth Content

Interviews and structured participant observations from samples 1 and 2 produced verbal and visual data that were analyzed by the principal investigator. Interviews were the primary source of the research findings, whereas observations were used to confirm the data collected from the interviews and to show how participants engaged with their mHealth technology.

Transcribed interviews were read several times, and deductive-inductive thematic analysis was undertaken using Atlas.ti software [33,34]. The stages of engagement and attributes from the framework by O’Brien and Toms [12] were applied to the interviews and observations. Deductive analysis was used to evaluate the conceptual framework, and a general inductive analysis was used to discover new attributes and important themes based on attributes related to each stage of engagement [35]. When a priori attributes and stages of engagement were not present in the data, they were removed; when new ones emerged, they were added to the schema. To reduce researcher subjectivity bias, 2 experienced health engagement researchers completed inter-rater reliability checks on the data. The inter-rater reliability calculated using the Cohen kappa coefficient was 0.92 in SPSS Statistics 26 (IBM Corp). This result suggests a strong level of agreement between the coders. Finally, mHealth engagement data through face-to-face observations confirmed participant use, thus triangulating the engagement states through both interviews and observations.

To establish message content, the occurrence and salience of themes at each engagement stage were established. Increased repetition of a theme at an engagement stage suggested increased salience of the corresponding message content. Thus, the most common themes and codes at each stage of engagement were identified and arranged in a hierarchy of the most relevant to be used for personalized communication.

## Results

### Participants

A total of 19 participants attended in-depth confidential interviews and structured participant observations using their mHealth technology with a researcher in sample group 1 (individual context) at the first period. Of the 19 participants, 12 (63%) were females and 7 (37%) were males, with age range of 18 to 49 years. The participants were invited to be interviewed and observed at 3 periods. At the second period, of the 19 participants, 15 (79%) participants completed the interviews and observations, with 11 (58%) participants agreeing to a third interview. In sample group 2, (group context) 25 participants were recruited from the GCC. Of the 25 participants, 18 (72%) were females and 7 (28%) were males, with an age range of 18 to ≥70 years. Again, each of the participants was invited to partake in the 3 periods. In total, 96% (24/25) participants completed both the second and third periods of data collection. Full participant details for sample 1 and sample 2 are provided in Table 1. Examples of technologies used across both samples included Fitocracy, Strava, Garmin Watch, Fitbit, or simple pedometers, and these were accessed via smart watches, mobile phones, and advanced trackers.

**Table 1:** Participant details for sample 1 and sample 2.

Participants	Samples	Gender	Age groups (years)	Length of time participants had been using their digital health technology at start of their interviews
1	Sample 1	Female	40-49	3-6 months
2	Sample 1	Female	30-39	6 months to 1 year
3	Sample 1	Female	18-29	<1 month
4	Sample 1	Male	40-49	1-3 months
5	Sample 1	Male	18-29	2 years
6	Sample 1	Female	18-29	1-3 months
7	Sample 1	Male	40-49	2 years
8	Sample 1	Female	40-49	2 years
9	Sample 1	Female	18-29	1-3 months
10	Sample 1	Female	40-49	1-3 months
11	Sample 1	Male	30-39	6 months to 1 year
12	Sample 1	Female	18-29	6 months to 1 year
13	Sample 1	Female	18-29	6 months to 1 year
14	Sample 1	Male	30-39	6 months to 1 year
15	Sample 1	Male	18-29	6 months to 1 year
16	Sample 1	Male	30-39	2 years
17	Sample 1	Female	40-49	1-3 months
18	Sample 1	Female	40-49	6 months to 1 year
19	Sample 1	Female	30-29	1 year
1	Sample 2	Female	30-39	3 years
2	Sample 2	Female	30-39	2 years
3	Sample 2	Female	30-39	1 year
4	Sample 2	Male	50-59	4 years
5	Sample 2	Female	18-29	1 year
6	Sample 2	Female	50-59	1 year
7	Sample 2	Female	50-59	1 year
8	Sample 2	Female	40-49	1 year
9	Sample 2	Female	60-69	>6 years
10	Sample 2	Female	50-59	2 years
11	Sample 2	Female	40-49	1 year
12	Sample 2	Male	50-59	3 years
13	Sample 2	Male	30-39	2 years
14	Sample 2	Female	30-39	2 years
15	Sample 2	Male	40-49	1 year
16	Sample 2	Female	40-49	2 years
17	Sample 2	Female	30-39	2 years
18	Sample 2	Male	50-59	3 years
19	Sample 2	Female	30-39	3 years
20	Sample 2	Female	30-39	1 year
21	Sample 2	Male	40-49	3 years
22	Sample 2	Female	50-59	4 years
23	Sample 2	Male	≥70	4 years
24	Sample 2	Female	40-49	4 years
25	Sample 2	Female	50-59	2 years

### Engagement Stages

The framework by O’Brien and Toms [12] explored engagement in the context of web searching, web-based shopping, educational webcasting, and video games, finding 4 stages of engagement: point of engagement, period of engagement, disengagement, and re-engagement. Similarly, sample group 1 identified these 4 types of engagement plus a fifth engagement domain of *self-management*. Self-management refers to a stage where users had completely disengaged from their technology; however, they were still engaged with their health activity.

**Figure 2:**
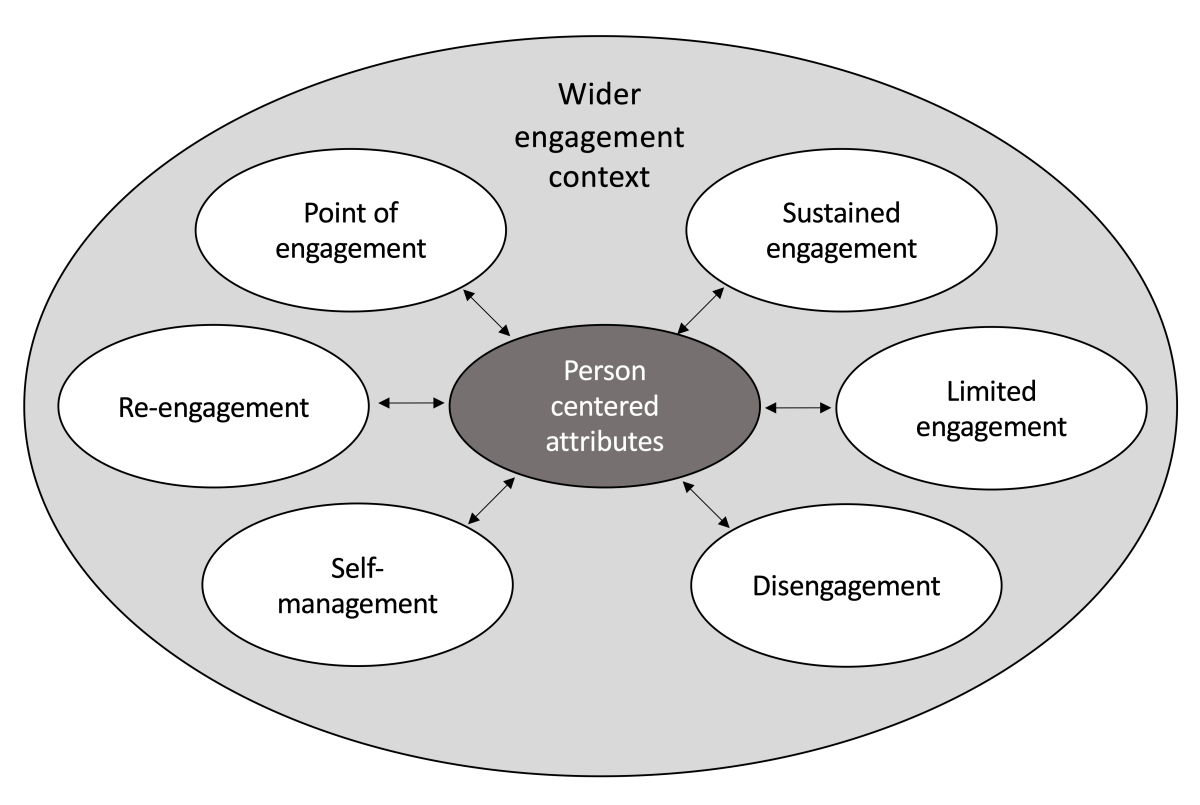
Mobile health engagement stages in this study (based on O’Brien & Toms 2008).

Furthermore, sample group 2 indicated an additional stage alongside self-management called *limited engagement*. This occurred when users had minimal interaction with their mHealth technology but engaged in the health activity to contribute to the group goals. Participants did not want to fully disengage as they felt the desire to stay in the program for others. Although limited engagement was discovered in the group context, it may also apply to solo engagement contexts. Thus, this research extends the framework by O’Brien and Toms [12] to include 6 stages of mHealth technology engagement, as seen in Figure 2. Importantly, these stages are not linear, with participants skipping stages based on motivation and accountability to engage, which are all features of a person’s wider engagement context.

### Themes at Each Engagement Stage

**Figure 3:**
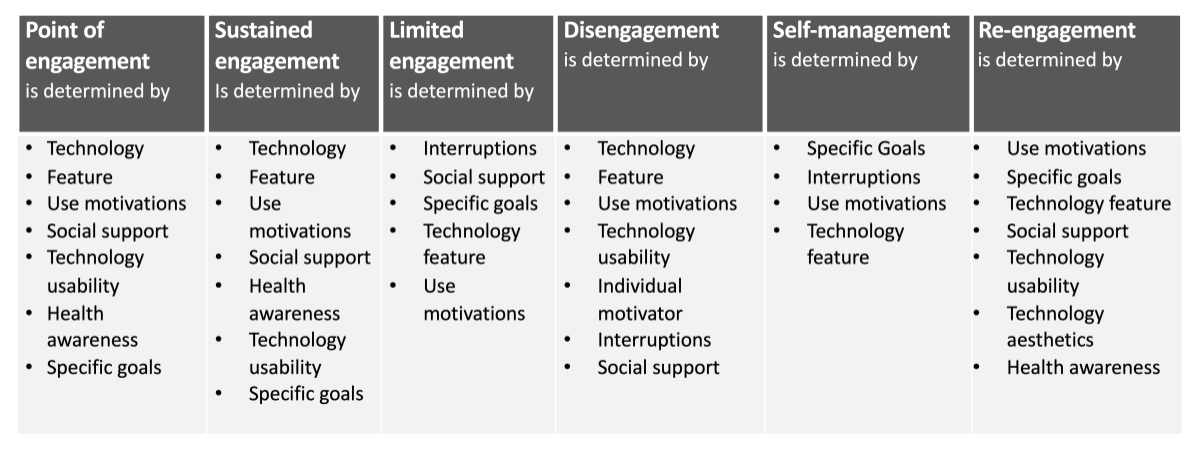
Themes relevant at each engagement stage.

When developing message content for sustained engagement, this research determined 8 themes that represent the main discussions impacting the 6 engagement stages. Each engagement stage is characterized by a different combination of these themes and 49 attributes. Figure 3 indicates the themes relevant at each engagement stage. These themes are presented in a hierarchical order based on their level of importance for each engagement stage. For example, in the limited engagement stage, the 5 themes of interruptions, social support, specific goals, technology features, and use motivations contribute toward shaping the level of limited engagement for the participant. The most frequently discussed theme for limited engagement was interruptions, with the lowest being use motivations. This indicates that interruptions are the greatest consideration for users experiencing limited engagement with mHealth technology. Multimedia Appendix 2 provides a full summary of the themes as they are expressed at each engagement stage.

On the basis of Textbox 1 and Multimedia Appendix 3, the point of engagement is characterized by the user’s attraction to the technical features of an mHealth device that helps them to fulfill their motivations to be healthy. At this stage, it is important that the technology is easy to use and promotes the support of a provider or a peer network. There was recognition that the mHealth device could improve users’ health awareness and their ability to reach their goals; however, these are less important than the features of the technology. Technology aesthetics was not highly relevant at the point of engagement.

**Mobile health engagement stages and themes characterizing each stage.**

- Point of engagement is characterized by technology feature, use motivations, social support, technology usability, health awareness, and goal setting
- Sustained engagement is characterized by technology feature, use motivations, social support, health awareness, technology usability, and goal setting
- Limited engagement is characterized by interruptions, social support, goal setting, technology feature, and use motivations
- Disengagement is characterized by technology feature, use motivations, technology usability, interruptions, and social support
- Self-management is characterized by goal setting, interruptions, use motivations, and technology feature
- Re-engagement is characterized by use motivations, goal setting, technology feature, social support, technology usability, technology aesthetics, and health awareness

Sustained engagement is characterized by the regular continued use of the mHealth device to support health and wellness activities. During this stage, technology features are important to achieve motivation, and this can be amplified through the support of a peer network or through provider messages. The continued use of an mHealth device encouraged users to have more health awareness. Usability and goal setting are still important in this stage but are less important than the features of the technology.

Limited engagement is characterized by users’ interrupted use of the mHealth device, with participants driven by social motivations to continue with the health activity. Social support through a peer network or provider messages was considered by users as influential during this period to achieve their health goals. Interruptions experienced by users were often caused by a feature of the technology; however, social interactions provided the motivation to continue.

Disengagement is characterized by discontinuing the use of an mHealth device and participants stopping the health activity. This was often caused by the mHealth technology not being suitable for the user’s needs and not providing motivation to continue. This mainly occurred because the mHealth device lacked features or was difficult to use. In addition, users did not receive the required social support from their device to develop sustained engagement. This was because of the inability of the mHealth technology to engage the user’s peer network or support provider messages. Finally, other interruptions also caused disengagement.

Self-management is characterized by users who are able to stay motivated and achieve their goals without their mHealth technology. Interruptions caused by the technology features meant that an mHealth device was not appealing and no longer used. In addition, as users were already active and aware of their health status, they did not need their mHealth technology for health awareness.

Re-engagement is characterized by users wanting to be motivated and wanting to achieve their goals. During this period, the technology features and technology aesthetics or the visual appeal of a device were important in the decision to re-engage. When re-engaging with existing or new technology, users were after specific technology features to help reach their health goals (eg, heart rate monitor and step counter). In addition, users were looking to re-engage with a mHealth technology that provides social support and helps them achieve health awareness.

### Description of Each Theme

A total of 8 themes were established in interviews across the 2 samples: technology usability, features, technology aesthetics, use motivations, health awareness, goal setting, social support, and interruptions when engaging with mHealth technology. To develop message content, an understanding of the themes, their attributes, and the relevant stage of engagement is considered below.

#### Technology Usability

The usability of the mHealth wellness technology was discussed by most participants and was important at the points of engagement, sustained engagement, disengagement, and re-engagement stages. Fundamentally, this was related to functional congruence or the device performing in a way that was required by the wearer [36]. This finding is consistent with other studies that highlight the importance of usability [13,14]. Usability was specified as ease of use, wearability, portability, convenience, and connectivity. In addition, accuracy and customization were important features when participants engaged with mHealth technology at the point of engagement and to a lesser extent once they were engaged. Disengagement occurred when the device did not perform as expected; it could not be customized, resulting in a nonpersonalized experience or a feature of the technology failed. Many participants lamented that if they could not see their previous activity, this reduced motivation and increased effort to re-engage:

I like that it’s simple to use, you just literally put it on your arm, load up on to your phone and just go, you don’t have to do anything with it.

Participant 9: female, aged 18-29 years, sample 1

I like how the GCC and the Fitbit thing they sync with that device...I like that seamless syncing so that it already knows what’s happened.

Participant 20: female, aged 30-39 years, sample 2

#### Technology Feature

The most relevant topic across both samples was the features of the mHealth technology, which was discussed by all participants and was present in each stage of engagement. Participants found value in the collection, monitoring, and review of health data, with it providing motivation to engage and use mHealth technology. Indeed, user-generated health data have been recognized to engage and empower patients in health care [37], and this also occurred in the wellness context. Gamification, messages, sounds, and dashboard tracking were features of devices that promoted continued use and the statistics provided through the Fitbit. However, if interruptions occur, for example, by a device having a short battery life, it could lead to disengagement and subsequent self-management. Although motivation to achieve goals was the predominant reason for re-engaging with technology, technology features also impacted the decision to re-engage:

...data, that was what led me to get this one because I wanted my heart rate and it gave me that and it gives me whatever data I want really on my sleep or my heart rate or exercise patterns, it can give me as much or as little as I want.

Participant 1: female, aged 40-49 years, sample 1

#### Technology Aesthetics

Despite the emphasis on design elements in the extant literature [14,15], this was only a minor theme discussed in interviews by one-third of the participants. Defined as an attractive interface, ergonomic design, and unobtrusive when worn, these attributes were only relevant at the re-engagement stage. Disengagement because of failure of a product feature affected future mHealth technology choices, and new offerings with improved design were considered to supersede predecessors. This was most evident in sample 1, where participants used the devices alone, whereas in sample 2, they were much more likely to re-engage based on perceptions of usability:

The new one is a bit thinner I think, this isn’t too bad, I think it can still do the same as heart rate and all of that, but then I suppose after a while I might end up getting a thinner version.

Participant 6: female, aged 18-29 years, sample 1

#### Use Motivations

Although technology features were the most important aspect of mHealth adoption, motivation to use the technology was a heavily discussed theme at every stage of engagement. Motivation was considered key to sustained engagement, and although it is accepted that individual differences in motivational control exist [38], people generally have individual intrinsic motivators in sample 1, and in sample 2, extrinsic social motivators became more relevant. Being active, reducing stress, and having a balanced lifestyle were motivations to use an mHealth technology in the pursuit of achieving wellness and a long life. When using technology in sustained engagement, motivation was derived through technology features via encouragement to achieve challenges, competition through leader boards, and the sense of team obligations. In addition, achieving health goals left people feeling good about their technology experience and encouraged sustained use and re-engagement:

After the baby I wanted to get fit again and feel good about myself so either way I was going to be doing it. I just wanted something there, I could go back and have a look and see my fitness improving over time not just having to guess a particular thing.

Participant 3: female, aged 18-29 years, sample 1

...and on Facebook as well, we’ve got a chat group in messenger because all in our group are friends on Facebook, so sometimes we message each other on the weekend and say this morning I got 10,000 steps already or something like that. I think that when everyone else in the group sees that they feel like I’d better do something myself.

Participant 15: male, aged 40-49 years, sample 2

#### Health Awareness

Awareness was an attribute in the framework by O’Brien and Toms [26] and was prevalent in interviews, becoming the theme, health awareness, in this research. Engaging with an mHealth technology that results in greater awareness of health through the collection and analysis of health data is consistent with the quantified self-movement, defined as *self-knowledge through numbers* [39]. The theme was evident at the point of engagement and became more relevant in sustained engagement and was referenced again in re-engagement. Participants were motivated at the point of engagement by awareness of health and wellness goals and could objectively evaluate their performance when using the technology during sustained engagement, with re-engagement partly spurred by the awareness of previous health outcomes. This led to enhanced behavior recognition and increased personal accountability for health activities. This theme was not important at limited engagement, disengagement, or self-management, as these stages suggest low exposure to data collection and analysis:

So, I can actually see that I’m more active than what I originally thought because I thought I only do about 30 minutes of walking on the treadmill or whatever but now I’m realizing I’m doing an extra 6,500 steps.

Participant 3: female, aged 18-29 years, sample 1

#### Goal Setting

Although goal setting was noted at the point of engagement, sustained engagement, and limited engagement, it became the most relevant theme in self-management and important for re-engagement. Participants spoke of goals related to exercise, nutrition, sleep, weight, and heart rate before and during sustained use of the mHealth technology, although these were less important than technology features, use motivations, and technology usability. As technology was not present in self-management, goals became the focus of wellness activities. Finally, goals were an avenue to re-engage with their existing or new mHealth technology, with participants having more sensitivity to goals after their first experiences. This was because of an increased awareness of devices, the ability to self-quantify, and the previous experience of achieving goals:

It’s simple to use and there’s a lot of scope to how I can plan a run. I can get it to remind me every pretty much time, calories, the goal I reach.

Participant 15: male, aged 18-29 years, sample 1

...and I want to be fabulous at 50, so I thought this will encourage me to do a few extra steps and think about what I’m eating, so that’s my goal for November.

Participant 5: female, aged 18-29 years, sample 2

#### Social Support

Social support was expressed in sample 1 as customer support and sample 2 as peer support. This was most relevant at the point of engagement, sustained engagement, limited engagement, and re-engagement. At the point of engagement, participants contacted customer support to understand a device feature for better use. Social support was relevant during sustained and limited engagement, including discussion of team interactivity and building a sense of community. Peer competition was an extrinsic motivator to do better, and when group goals were achieved, it enhanced the profile of the teams in the wellness community. When self-management was present and devices were no longer used, social support became less relevant; however, when re-engaging with technology, previous experiences of a social support community were influential in the uptake of new devices:

Maybe it’s not so much to do with Strava but maybe it’s just that there’s a community of people that helps facilitate, maybe it’s something to do with just that people can see what others are up to and follow them and share in their experiences.

Participant 16: male, aged 30-39 years, sample 1

I like the team. I like that idea of people being in a team together contributing, motivating each other.

Participant 16: female, aged 40-49 years, sample 2

#### Interruptions

Interruptions were a critical element in limited engagement, disengagement, and self-management stages. Interruptions were caused by an individual’s engagement. Family factors, work time constraints, and illnesses impacted limited engagement. Technology failure included losing a device or being unable to access data, and this impacted disengagement and the decision to self-manage. Device features were also relevant, with low functionality resulting in a perception that devices were not holistic, leading to limited use and disengagement. Indeed, disengagement can occur in the limited engagement stage, even in the presence of social support, if an individual experiences significant interruptions:

Unfortunately, the battery goes very, very quickly and then these times where I can’t hold the phone because I’m doing stuff so it’s not monitoring my steps.

Participant 12: female, aged 18-29 years, sample 1

I think for me with kids I needed a program where I can go back three days and log what I’d done because I can get distracted or sidetracked.

Participant 3: female, aged 18-29 years, sample 1

#### Developing Message Content

Multimedia Appendix 3 illustrates the full list of 49 attributes that emerged from the research relevant to each of the mHealth themes. These attributes highlight the key factors that determine the expression of each theme. By identifying the themes important at each engagement stage and then the relevant attributes, health care providers and mHealth developers can determine the type of content in messages that aim to sustain engagement.

For example, at the point of engagement, the most important theme that emerged was the technology feature. The attributes related to this theme are health data collection, dashboard tracking, messages, sounds, and device battery life. If health care providers such as insurers or personal trainers want to attract clients, the content of messages could focus on technology features such as the ability to track the user’s data (dashboard tracking) or the use of health information (health data collection). When users demonstrate limited engagement, messages can focus on the challenges of interruptions to health programs, such as competing the demands of family and providing some tips for how they can manage these difficult times. If users show re-engagement behaviors, messages could focus on reminding users of personal motivations for health management, focusing on specific small goals they may want to achieve.

**Figure 4:**
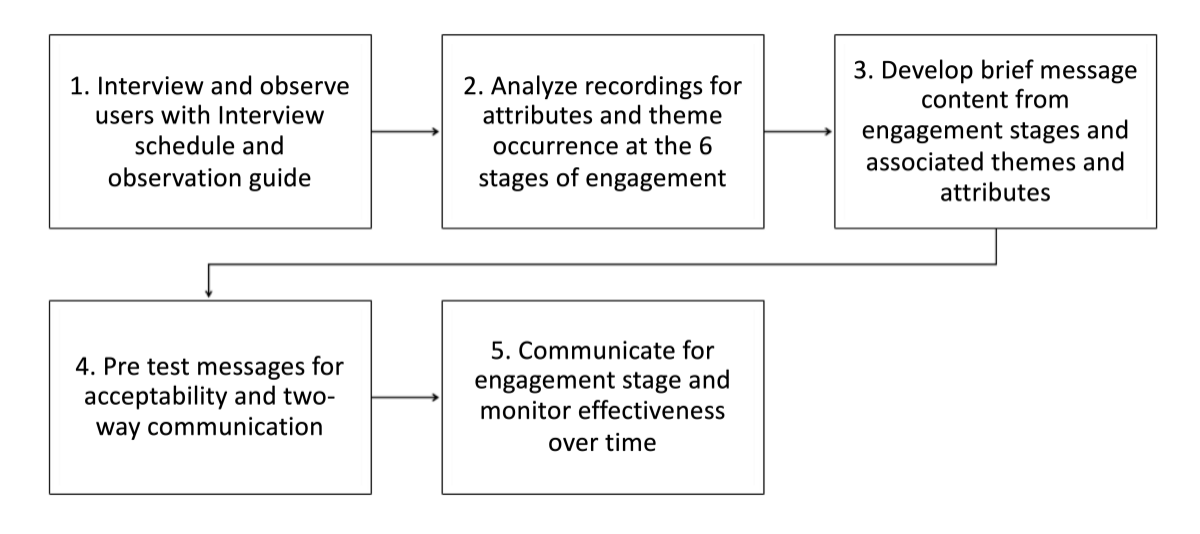
A five-step process to develop effective brief message content.

Therefore, with engagement stages defined in this research and identification of key themes and attributes shaping people’s engagement over time, this research recommends a 5-step process to help co-design appropriate content around these engagement stages for mHealth users. This co-designed 5-step process will help ensure that messages are meaningful with the aim of improving mHealth device engagement over time (Figure 4).

The 5-step process highlights the importance of first understanding the components necessary to group users into relevant engagement stages. By interviewing and observing users (step 1), organizations can identify which of the 6 engagement stages the mHealth user is at (step 2). This allows targeted communication relevant to the individual and their engagement stage to be developed, encouraging sustained engagement (step 3). The relevance of the message content can be drawn from the attributes identified. Step 4 involves pretesting the messages for acceptability by the user and to ensure the development of a two-way communication between the health care provider and the user. Without ensuring the suitability and targeting of the message content, health care providers run the risk that the target user will not pay attention to the message [40]. The final stage (step 5) involves the delivery of suitable messaging representative of the engagement stage the user is at, including monitoring the message success. This monitoring could come in the form of interactive responses via text messages, rebooking with health care staff, user-generated content entered into an app, or other such responses indicating engagement. This process is applicable across a broad application of mHealth relevant contexts, including but not limited to wellness initiatives and clinical contexts; however, programs require targeting and tailoring to an individual’s context to ensure maximum communication success.

## Discussion

The use of mHealth technology for patient engagement is enhanced by communication between health care providers and the technology user. To date, an engagement framework that can be flexibly applied in different contexts to develop message content has been under-researched in the extant literature. This research presents a model of 6 engagement stages: point of engagement, sustained engagement, limited engagement, disengagement, self-management, and re-engagement. Stages are characterized by 8 themes and 49 attributes, which signify the total user experience.

Importantly, the 6 engagement stages are not linear and have been reconceptualized using a person-centered approach, taking into account a user’s wider engagement context. In a group context, mHealth devices can instigate limited engagement where the extrinsic motivation of the group performance is the only reason to engage. If limited engagement is recognized, however, message content can be tailored to increase the intrinsic motivation of goal setting to achieve sustained engagement. Similarly, after episodic wellness initiatives such as the GCC cessation, participants can still be engaged in health activities without the technology present. To pique interest and re-engagement in devices, technology aesthetics and features can be communicated to ex-users. Finally, people who disengage because of interruptions and find it difficult to re-engage may benefit from motivating message content that promotes limited engagement and social support as a goal before sustained engagement.

This research extends previous investigations into engagement stages, as initially proposed by O’Brien and Toms [12]. Although previous research has investigated the stages of engagement from a business and information systems perspective [12], this study is one of the first investigations to specifically explore and extend these stages for an mHealth context. Within a health context, where the stages of engagement proposed by O’Brien and Toms [12] have been applied, there have been no developments in the existing stages or attributes, acknowledging the unique context [29,30]. Keeling et al [11] are the only researchers to further develop the stages proposed by O’Brien and Toms for a web-based health context; however, these researchers focused on investigating into digital engagement to understand why users do not engage with web-based health services. Although these researchers have contributed to knowledge about disengaged users, they observed that further research is needed into mHealth apps, as health conditions alter with time and research into touchpoints over time would be advantageous to better understand engagement. This study, to an extent, fulfills these shortcomings identified by Keeling et al [11].

The development of brief message content for mHealth engagement is a nascent research area, with existing studies focusing on specific providers or medical conditions. Indeed, many studies approach message development by creating content that emphasizes the providers’ needs, rather than taking a person-centered approach, which involves understanding the wider context for the user [41], as undertaken in this research.

The standard approach to develop brief message content for mHealth is to align message content with an appropriate theory, develop content based on a desired behavior, and then pretest the messages on users, often rating acceptability using a validated survey [42]. Although message pretesting is considered a priority to “understand and learn about the specific audience’s preferences in technology use, language, and health needs” [40], co-designing message content with end users is critical for acceptability and workflow integration [43]. Methods to create targeted messages for mHealth engagement using a person-centered approach are limited, although there is recognition that researchers need to consider formative qualitative investigation with the target population before message development [44], as was conducted in this research.

This study extends the shortcomings of previous research by developing a 5-step process to develop co-designed message content [40]. The attributes, themes, and engagement stages identified in this study can be used to develop personalized two-way message content that can be verified by message pretesting. If a health care provider, such as a weight loss clinic, wants to focus on a user who is demonstrating behaviors associated with the disengagement stage, they can ask about interruptions in brief message content and encourage re-engagement with an existing or new device with different features to achieve health goals. Attributes that are important to goal setting may include exercise, nutrition, sleep, weight, or heart rate. The proposed process highlights the importance of co-designing messages to ensure the best response and acceptance by users.

### Conclusions

On the basis of current evidence, brief messages are cost-effective and can be personalized and delivered at scale in multiple settings to improve sustained health engagement. This research demonstrates that end-user mHealth engagement is complex, nonlinear, can be social or solo, and is characterized by 6 stages. Each stage is defined by themes and attributes that signify the total user experience. Fundamentally, this research calls for the consideration of interdisciplinary frameworks that incorporate health, information systems, and business to avoid the partial view of this phenomenon and to enhance engagement over time.

The limitations of this research are the small sample size from a single first-world country. Future research could measure the existing, and explore for additional, engagement stages using a larger sample and in different cultures. This would allow for more detailed investigations between groups, such as those users at different stages in the engagement journey, those displaying different demographic characteristics, or those using different types of technologies.

Further research could also include larger quantitative investigations that could be used to overcome the more exploratory and perhaps limited representative sample in this study. Future quantitative research could investigate interventions at each engagement stage or use experimental design to test the suitability of message content across these stages. The development of a short identification survey could also be undertaken, focusing on the 6 engagement stages. This would enable quick categorization of users and the stage they are at in their engagement journey, thus limiting the reliance on more longer time-intensive interviews for organizations that could result in more easily obtained targeted communication.

## Appendix

Multimedia Appendix 1 Interview and observation protocol.

Multimedia Appendix 2 Expression of each theme at each engagement stage.

Multimedia Appendix 3 Brief communication content attributes.

## Abbreviations

GCC: Global Corporate Challenge
mHealth: mobile health
