# Supplementary material for: Identifying Mobile Health Engagement Stages: Interviews and Observations for Developing Brief Message Content
Source: J Med Internet Res. 2020 Sep 22;22(9):e15307. doi: 10.2196/15307 (PMC7539166; doi:10.2196/15307)
Supplement: Multimedia Appendix 1 [file jmir_v22i9e15307_app1.docx]

## Multimedia Appendix 1. Interview and observation protocol.

| Interview Protocol |
| --- |
| 1. Can you tell me about your perception of wellness? |
| 1. Why did you engage with an online wellness program? |
| 1. How have you been using the program? |
| 1. What parts of the program are you using? |
| 1. What parts of the program are you not using? |
| 1. When do you use the program? |
| 1. How often do you use the program? |
| 1. What is your experience when using your online wellness program? |
| 1. If you use a pedometer what is your experience when using the pedometer? |
| 1. Do you receive notifications? When do you interact with notifications? |
| 1. Do you use any social media to upload, communicate or view other people’s achievements? |
| 1. Has the program impacted your behavior towards wellness? |
| 1. What do you expect to gain from your participation in this program? |
| 1. Can you tell me about a positive experience when using the online program? |
| 1. Can you tell me about a negative experience when using the online program? |
| Observation Protocol |
| 1. Can you please show me what you do first when you use the website? |
| 1. Can you please show me what parts of the website you use? |
| 1. Can you please show me what parts of the website you don’t use? |
| 1. Can you please show me what you find useful? |
| 1. Can you please show me how you communicate with others while engaging with this service? |
| 1. What is missing? Is there anything that you would like to be able to do that the website does not support? |
