# Supplementary material for: Identifying Mobile Health Engagement Stages: Interviews and Observations for Developing Brief Message Content
Source: J Med Internet Res. 2020 Sep 22;22(9):e15307. doi: 10.2196/15307 (PMC7539166; doi:10.2196/15307)
Supplement: Multimedia Appendix 2 [file jmir_v22i9e15307_app2.docx]

## Multimedia Appendix 2. Expression of each theme at each engagement stage.

|  | **Point of engagement** | **Sustained engagement** | **Limited engagement** | **Disengagement** | **Self-management** | **Re-engagement** |
| --- | --- | --- | --- | --- | --- | --- |
| **Goal**  **setting** | Users had a desire to achieve a specific goal that they believed that their health technology could help them to reach their goal. | Users found their digital health technology was helping them to reach their goals, keeping them motivated to continue to use and achieving sustained engagement. | Users did not feel that their digital health technology was enabling them to reach their health goals and only intermittently used the technology, driven by other motivations. | Users found reaching their goals was challenging and/ or their digital health technology was not suited to their needs. Their digital health technology was not helping or motivating them to achieve their goals. | Users found that they were able to achieve their goals without their digital health technology. They could effectively self-manage to achieve their goals. | Users were looking to re-engage with their previous digital health technology or re-engage with a different digital health technology to assist them with their needs for reaching renewed health goals. |
| **Technology**  **feature** | Users were attracted to specific digital health technologies that provided features that they would be interested in engaging with (e.g., step tracking, heart rate tracking, etc.). | Users were happy with the features that their digital health technology offered. The technology features helped them to reach their goals for sustained engagement. | Users found that their current digital health technology feature was not suiting their needs or requirements. As a result, the digital health technology was not being used to its full potential and alternatives were sought. | Users found that their digital health technology did not meet their needs. This could either be because their digital health technology was lacking features that users were after. | Users found that the technology was no longer appealing enough for them to continue using and they could self-manage on their own. Either the features were not appealing, or the user was after other features digital health technology features. | When re-engaging with a new technology or their existing technology, users were after specific technology features to help reach their health goals (e.g. the user wants a heart rate monitor, step counter, GPS etc.). |
| **Use motivations** | Users are looking for a digital health technology that will help to motivate them to achieve their goals. Motivating features include feedback, tracking, rewards and competing with others. | Users were motivated to use their digital health technology and self-health manage. Their digital health technology is providing them with the motivation required to achieve sustained engagement. | Users found that the digital health technology was not providing them the motivation to consistently use the digital health technology and or reach their health goals, often due to lack of features. | Users lacked motivation to want to use their digital health technology and often decided to self-manage. The digital health technology was not engaging enough and was not providing them the motivation they required. | Users were motivated to self-manage without their digital health technology. They are able to motivate themselves without an external digital health technology force. | Users re-engaged with a digital health technology that helped motivate them to reach their health goals. This was driven by features and the aesthetics of the health technology. |
| **Health awareness** | At the point of engagement, users were looking for something that would make them person aware and assist them to self-manage their health goals. Self-tracking and comparing their tracking data to others was seen to increase health awareness and motivation to achieve goals. | Users found the digital health technology was making them aware of their current health state and the improvements as they used the health technology. Due to this awareness, users improve their health while continuing to use their digital health technology, achieving sustained engagement. | Users lacked awareness of the connection between using the digital health technology and achieving their health goals. This could be because the digital health technology is not challenging enough for them, they had become used to the technology and lacked motivation to stay aware, or that the user does not use the digital health technology frequently enough to become more aware of the connection between their health and technology use. | Users did not find that the digital health technology made them aware of their health. This could be because the digital health technology was not challenging or motivating enough for them, or the user did not use the digital health technology to become aware of their own health. | Users were already active and aware of their current health state. They did not need their digital health technology and can self-manage on their own health. | Users re-engaged with their old or new digital health technology to make them more aware of their health status. |
| **Social support** | Users were looking for a specific type of support while engaging in their digital health technology. This could be through family, friends or through social support in the digital health technology. | Users found they were getting the required support from their digital health technology, friends, and/or family which helped them stay motivated to reach their health goals, achieving sustained engagement. | Users lacked the required support gained through their digital health technology. The digital health technology may not be providing enough support if there are interruptions to using the device and users may not be receiving enough notifications and encouragement from their digital health technology. | Users were not getting enough support from their digital health technology, family, or friends to want to keep engaging with their digital health technology. The digital health technology may not engage the user’s friends or family to support the user to help reach their health goals. | For users, social support was no longer needed when engaging with their digital health technology as they can self- manage on their own. Some users felt pressure from their social support, which discouraged them to continue engaging. | Users were looking to re-engage with a digital health technology that provides social support and helps them on their health journey. This could be through notifications. The user may also want to connect their device up with their friends and family for further support. |
| **Technology usability** | Users are looking for a digital health technology that is easy to use and understand. | Users enjoyed having an easy to use digital health technology. This kept them engaging with their digital health technology and improved the understanding of how to use all the features of the technology. | Users had issues understanding how to use their digital health technology. This led to the user only engaging on a limited basis with their digital health technology. | Users has issues with the usability of their digital health technology. They found it frustrating, making it difficult for them to remain engaged. | N/A | Users are looking for a new technology or will only re-engage in their existing technology if it is easier to use. Some users looked up reviews prior to purchasing or using the digital health technology. |
| **Technology aesthetics** | Users were attracted by the aesthetics of their digital health technology which encouraged them to interact and engage with their digital health technology. | Users liked the aesthetics of their digital health technology. This helped them to remain at a sustained level of engagement. | Users felt that the aesthetics of their digital health technology did not meet their requirements and were discouraged to reach a level of sustained engagement with their digital health technology. | Users felt that the aesthetics of the digital health technology did not meet their requirements. This caused them to disengage with their digital health technology. | N/A | Users were looking for particular aesthetics aspects that would be appealing for them to re-engage in a digital health technology. |
| **Interruptions** | N/A | N/A | Users faced problems while using their digital health technology which led to limited usage. There could also be personal problems not associated with the digital health technology (e.g. getting sick, family issues, being busy etc.). | Problems not associated with the digital health technology (e.g. getting sick, family issues, being busy etc.) or with the technology itself caused users to disengage. Problems not associated with the digital health technology became a higher priority for users to focus on at the time. | Despite users facing problems that impacted their digital health technology usage, some did continue to self-manage without using their digital health technology. | Even though users wanted to re-engage in a new digital health technology, users had encountered personal problems during this process or with the digital health technology itself. |
