# Supplementary material for: Identifying Mobile Health Engagement Stages: Interviews and Observations for Developing Brief Message Content
Source: J Med Internet Res. 2020 Sep 22;22(9):e15307. doi: 10.2196/15307 (PMC7539166; doi:10.2196/15307)
Supplement: Multimedia Appendix 3 [file jmir_v22i9e15307_app3.docx]

## Multimedia Appendix 3. Brief communication content attributes.
